# Supplementary material for: Detailed molecular and epigenetic characterization of the pig IPEC-J2 and chicken SL-29 cell lines
Source: iScience. 2023 Feb 20;26(3):106252. doi: 10.1016/j.isci.2023.106252 (PMC10018572; doi:10.1016/j.isci.2023.106252)
Supplement: Data S2. Complete homer output for identified motifs in Chicken SL-29, related to Tables 5 and 6 — Homer motif analysis results for histone modifications H3K4me1, H3K4me3, H3K27ac, enhancers, and ATAC-seq of chicken SL-29 cell line. Parameters for possible false positives is as mentioned earlier for S5. [file mmc3.zip › Data_S2/S6/Chicken_SL_29/peak_files_CTCF/homerResults/motif5.similar.html]

motif5

## Information for motif5

C
G
T
A
T
C
G
A
T
G
A
C
G
T
A
C
C
T
G
A
A
C
G
T
T
G
C
A
A
G
C
T
A
C
T
G
A
C
G
T
  
Reverse Opposite:  

T
G
C
A
G
T
A
C
C
T
G
A
A
C
G
T
T
G
C
A
A
G
C
T
A
C
T
G
A
C
T
G
A
G
C
T
G
C
A
T
  

|  |  |
| --- | --- |
| p-value: | 1e-179 |
| log p-value: | -4.136e+02 |
| Information Content per bp: | 1.588 |
| Number of Target Sequences with motif | 7627.0 |
| Percentage of Target Sequences with motif | 25.87% |
| Number of Background Sequences with motif | 5545.3 |
| Percentage of Background Sequences with motif | 19.06% |
| Average Position of motif in Targets | 150.5 +/- 83.3bp |
| Average Position of motif in Background | 148.8 +/- 86.8bp |
| Strand Bias (log2 ratio + to - strand density) | 0.0 |
| Multiplicity (# of sites on avg that occur together) | 1.23 |
| Motif File: | file (matrix) reverse opposite |

### Similar de novo motifs found

|  |  |  |  |  |  |  |  |
| --- | --- | --- | --- | --- | --- | --- | --- |
| Rank | Match Score | Redundant Motif | P-value | log P-value | % of Targets | % of Background | Motif file |
| 1 | 0.958 | T G C A T G C A T G A C C T G A A C G T T G C A G A C T A C T G | 1e-143 | -331.369448 | 25.08% | 19.02% | motif file (matrix) |
| 2 | 0.875 | A G T C T G C A C A T G G T A C A G C T A C T G C G A T G A C T | 1e-120 | -277.600826 | 38.15% | 31.70% | motif file (matrix) |
| 3 | 0.874 | G C T A T A G C T G A C C G T A A C T G G T C A C G A T T C A G A G C T A G C T C G A T A C G T A G T C | 1e-116 | -269.065522 | 14.29% | 10.04% | motif file (matrix) |
